# Mobile Apps for the Management of Gastrointestinal Diseases: Systematic Search and Evaluation Within App Stores

**DOI:** 10.2196/37497

**Published:** 2022-10-05

**Authors:** Eva-Maria Messner, Niklas Sturm, Yannik Terhorst, Lasse B Sander, Dana Schultchen, Alexandra Portenhauser, Simone Schmidbaur, Michael Stach, Jochen Klaus, Harald Baumeister, Benjamin M Walter

**Affiliations:** 1 Department of Clinical Psychology and Psychotherapy Institute of Psychology and Education University of Ulm Ulm Germany; 2 Department of Internal Medicine I University Hospital Ulm Ulm Germany; 3 Department of Research Methods Institute of Psychology and Education University of Ulm Ulm Germany; 4 Department of Rehabilitation Psychology and Psychotherapy Institute of Psychology Albert-Ludwigs-University Freiburg Freiburg at Breisgau Germany; 5 Department of Clinical and Health Psychology Institute of Psychology and Education University of Ulm Ulm Germany; 6 Institute of Databases and Information Systems University of Ulm Ulm Germany

**Keywords:** gastrointestinal diseases, mHealth, mobile health, MARS, Mobile Application Rating Scale, systematic review, app quality, gastrointestinal, mobile app, app

## Abstract

**Background:**

Gastrointestinal diseases are associated with substantial cost in health care. In times of the COVID-19 pandemic and further digitalization of gastrointestinal tract health care, mobile health apps could complement routine health care. Many gastrointestinal health care apps are already available in the app stores, but the quality, data protection, and reliability often remain unclear.

**Objective:**

This systematic review aimed to evaluate the quality characteristics as well as the privacy and security measures of mobile health apps for the management of gastrointestinal diseases.

**Methods:**

A web crawler systematically searched for mobile health apps with a focus on gastrointestinal diseases. The identified mobile health apps were evaluated using the Mobile Application Rating Scale (MARS). Furthermore, app characteristics, data protection, and security measures were collected. Classic user star rating was correlated with overall mobile health app quality.

**Results:**

The overall quality of the mobile health apps (N=109) was moderate (mean 2.90, SD 0.52; on a scale ranging from 1 to 5). The quality of the subscales ranged from low (mean 1.89, SD 0.66) to good (mean 4.08, SD 0.57). The security of data transfer was ensured only by 11 (10.1%) mobile health apps. None of the mobile health apps had an evidence base. The user star rating did not correlate with the MARS overall score or with the individual subdimensions of the MARS (all *P*>.05).

**Conclusions:**

Mobile health apps might have a positive impact on diagnosis, therapy, and patient guidance in gastroenterology in the future. We conclude that, to date, data security and proof of efficacy are not yet given in currently available mobile health apps.

## Introduction

Gastrointestinal diseases are associated with substantial morbidity and health care costs worldwide [1-5]. For example, in the United States, the annual health care expenditures for gastrointestinal diseases were US $135.9 billion in total, with more than 54.4 million ambulatory visits with a primary diagnosis for gastrointestinal disease and 3.0 million hospital admissions [6]. Additional indirect costs arise due to substantial levels of personal disability, work absenteeism, and loss of productivity [7-12]. Therefore, health care systems are challenged to provide equitable and affordable solutions for patients with digestive diseases [6,13].

In particular, for the successful treatment of chronic gastrointestinal diseases (eg, inflammatory bowel disease [IBD] and irritable bowel syndrome), the patient’s adherence and compliance are crucial [14-19]. Treatment recommendations are extensive, consisting of medical and psychological measures [20-24]. Moreover, they include high-demand interventions such as health behavior changes (eg, dietary adjustments or stress management) that cannot be addressed adequately in routine health care [6,24-26]. Additionally, the COVID-19 pandemic with consecutive lockdown forced the health care institutions to uptake contactless approaches [27-32]. Therefore, the implementation of mobile health (mHealth) apps might be a promising approach [33-36].

A recent US study showed that 58.2% of smartphone users had at least 1 mHealth app downloaded on their device [37]. Fitness and nutrition apps were the most commonly downloaded mHealth apps [37]. However, mHealth solutions might also have a potential impact in prevention, diagnostics, and therapy in gastrointestinal disorders [38].

Unfortunately, there is a relevant gap between the high number of available mHealth apps to manage gastrointestinal diseases and the low number of reliable scientific studies in this field [33,36,39]. This gap is concerning as the use of mHealth apps is accompanied with potential risks and side effects such as insufficient data protection and a lack of privacy, as well as treatment without informed consent [40]. Other potential hazards such as misinformation, nonavailability in emergencies, and data misuse have been reported for mHealth apps [40,41].

Due to the rapid development in technology, users and health care providers have difficulties in identifying relevant, high-quality mHealth apps, because they have to rely on the information provided in the stores such as user star ratings and app descriptions [42]. Previous studies have already indicated that user star ratings are potentially misleading because they are influenced by user-friendliness and functionality rather than by content quality [43]. Furthermore, they might be biased due to fake ratings or older versions of the app [42-44]. Therefore, user star ratings might not be a valid orientation aid for selecting a mHealth app, and other strategies to support users and health care providers select an appropriate mHealth app to manage health care issues should be considered.

Additionally, many scientifically tested apps developed by universities and research projects do not enter the app market [45]. In contrast, many available mHealth apps developed by commercial providers have never been tested for their effectiveness and efficacy [45]. Therefore, the quality of publicly available mHealth apps for gastrointestinal diseases is not evident in the literature. Due to increasing public interest in the use of mHealth apps, reliable reviews and analyses are mandatory [46].

Quality-measuring instruments for mHealth apps such as the multidimensional Mobile Application Rating Scale (MARS) are available in several languages, validated, and used worldwide [47-50]. MARS is an expert rating tool that allows researchers to reliably assess and compare mHealth apps regarding user engagement, functionality, aesthetics, and the quality of information [50-52]. Furthermore, it offers a descriptive section in which aims, methods, theoretical background, and cost, etc, can be assessed [48,52]. The MARS was widely used to assess app quality systematically (eg, weight management, rheumatoid arthritis, chronic back pain, mindfulness, heart failure, chronic pain, posttraumatic stress disorder, medication adherence, depression, and smoking cessation, etc) [43,53-59].

The aim of this study was to systematically search for mHealth apps for gastrointestinal diseases in the app stores and evaluate their quality, content, and characteristics using the MARS [48]. Furthermore, mHealth app characteristics such as theoretical background, the content of the apps, affiliation, and price were assessed. Moreover, the accordance with gastroenterological guidelines and evidence base of the included mHealth apps were investigated.

## Methods

### Study Design

This systematic review was oriented on the PRISMA (Preferred Reporting Items for Systematic Reviews and Meta-Analyses) guidelines [60].

### Search Strategy and Procedure

An automatic search engine (Mobile Health App Database [MHAD] web crawler [61]) was used to systematically screen the Google Play and Apple App stores for eligible mHealth apps [62] between October 24, 2020, and June 12, 2021. The applied search terms were defined by conducting focus groups with patients with gastrointestinal disorders and health care providers at the University Hospital Ulm and Freiburg to mimic lay and professional searches. The final search terms included “digestive problems,” “stomach pain,” “constipation,” “CED,” “ulcerative colitis,” “Crohn’s disease,” “inflammatory bowel disease,” “reflux,” “bloating,” “diarrhea,” “celiac disease,” “food intolerances,” and “malabsorption.” The search terms were entered separately because logical operations and truncation cannot be used in the Google Play and Apple App stores.

All found mHealth apps were registered in a central database, and duplicates were automatically removed. All identified apps were screened regarding whether their title, description, given images, and comments of app users indicated that the app (1) was developed for gastrointestinal health issues, (2) provided in the German or English language, (3) was downloadable in the official Google Play or Apple App store, (4) was functional to enable an assessment (no device problems), and (5) met no other exclusion criteria (app bundles, only usable with another device such as a smartwatch, or not active for download). In a second step, the apps were downloaded and checked regarding the aforementioned criteria.

### Data Extraction, Evaluation Criteria, and Instruments

The included apps were evaluated by raters using the German version of the multidimensional MARS (MARS-G) [48]. Before starting with the evaluation process, the raters received standardized web-based training, which is publicly accessible and free of charge [63]. For quality assurance, interrater reliability (IRR) between the 2 raters was calculated. Rater agreement was examined by intraclass correlation (ICC) based on a 2-way mixed-effect model. A minimum ICC of .75 was predefined as sufficient ICC [64]. An additional reviewer was consulted when the IRR was below a value of .75 [48,64].

### Evaluation Tool MARS-G

The evaluation tool MARS-G is a reliable and valid procedure for the quality assessment of mHealth apps [48,52]. The MARS-G has a very good internal consistency for overall score (ω=.82, 95% CI 0.76-0.86) and high levels of IRR (2-way mixed ICC=.84, 95% CI 0.82-0.85) [48].

### General Characteristics

For examining app characteristics, the classification page of the MARS-G was used. It contains (1) the app name; (2) app version; (3) platform; (4) content-related subcategory; (5) store link; (6) price; (7) user star rating; (8) the number of user star ratings; (9) theoretical background (eg, type of therapy); (10) aims; (11) methods (eg, information/education, monitoring and tracking, gamification, and reminder); (12) technical aspects (eg, allows sharing); (13) data protection and safety (eg, password protection); (14) field of application; and (15) certification [48,50]. The classification site of MARS-G was used to assess the content and functions of the included mHealth apps [50,59]. With the MARS-G, a descriptive assessment of privacy and security features is possible. All features were assessed based on the information included in the mHealth apps or app stores. External information was not evaluated.

### Quality Assessment

The multidimensional quality rating of the MARS-G consists of 6 different subdimensions with 19 items, which can be evaluated on a 5-point Likert scale (1=inadequate, 2=poor, 3=acceptable, 4=good, and 5=excellent): (1) engagement (entertainment, interest, individual adaptability, interactivity, and target group); (2) functionality (performance, usability, navigation, motor, and gestural design); (3) aesthetics (layout, graphics, and visual appeal); and (4) information (accuracy of app description, goals, quality and quantity of information, quality of visual information, credibility, and evidence base); (5) subjective quality (recommendable, probability of using the app in the next 12 months, payment, and star rating); and (6) perceived impact (increased awareness, increased knowledge, attitudes, fosters intention to change, empowers help-seeking behavior, and fosters behavior change) [48,50]. For the assessment of the overall quality, the total score was calculated from the 4 main subdimensions (engagement, functionality, aesthetics, and information) [50]. The ratings of the reviewers were averaged for all calculations. Mean scores and SDs were calculated for the MARS overall score and subdimensions.

### Quality Rating on Evidence

To verify whether empirical studies were available for the mHealth apps, item 19 on the information subscale of the MARS was used. This item was examined by searching the mHealth apps’ name in Google, Google Scholar, PubMed, and the developers or providers’ website for existing efficacy and effectiveness studies [48].

### User Star Rating

The user star ratings were extracted from the app stores. The user star rating from Google Play and Apple App stores is rated on a scale of 1 to 5 stars. It is presented as a cumulative average of individual ratings in the app stores [65]. Pearson correlation coefficient between user star ratings and MARS-G ratings were calculated. For all analysis, an α level of 5% was defined [66].

## Results

**Figure 1:**
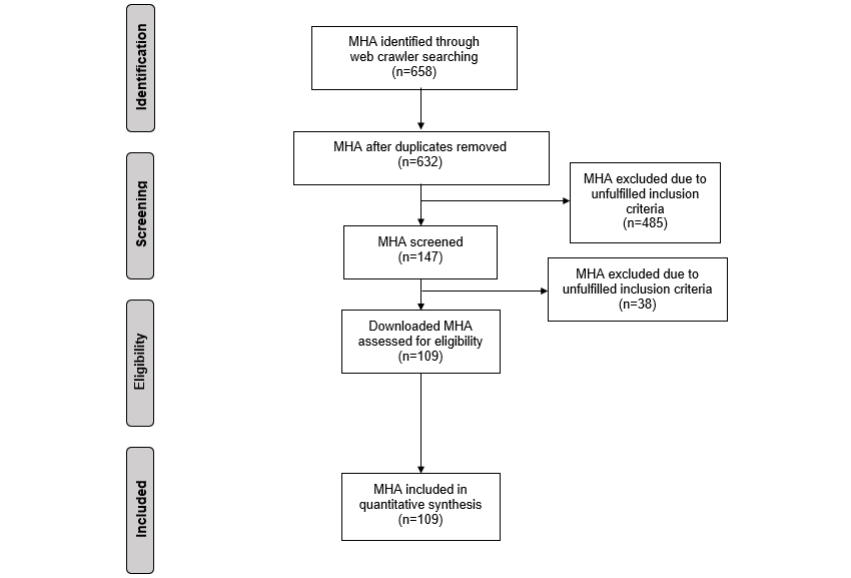
Flowchart of the inclusion process of mobile health apps (MHA).

The web crawler identified 658 mHealth apps, of which 109 were eligible for inclusion after screening and eligibility check (Figure 1).

### General Characteristics

Of the 109 mHealth apps, 79 (72.5%) were from the Google Play store, and 30 (27.5%) were from the Apple App store; 53 (48.6%) had a user star rating, whereas 56 (51.4%) were not rated by store users. The mean user star rating was 3.96 (SD 0.80), ranging from 2.00 to 5.00.

Most apps (n=93, 85.3%) were free of charge, and the prices of fee-based mHealth apps ranged from €0.69 to €8.99 (mean €4.0, SD €2.25; from US $0.84 to US $10.91; mean US $4.86, SD US $2.73). The 109 mHealth apps for gastrointestinal disorders were identified in the following Google Play or Apple App store categories (multiple categories can be assigned to 1 mHealth app): “health and fitness” (n=76, 69.7%); “medical” (n=33, 30.3%); “food and drinks” (n=11, 10.1%); “lifestyle” (n=3, 2.8%); “books and references” (n=2, 1.8%); “education” (n=2, 1.8%); “entertainment” (n=3, 2.8%); and “parenting” (n=1, (0.9%; Table 1).

The included mHealth apps targeted the following aims (multiple aims may be selected for 1 mHealth app): “improvement of general well-being” (n=92, 84.4%); “promotion of physical health” (n=86, 78.9%); “entertainment” (n=3, 2.8%); “support for behavioral changes” (n=33, 30.3%); “support in achieving individual goals” (n=27, 24.8%), “reduction of stress” (n=7, 6.4%); “reduction of fear” (n=4, 3.7%), “improvement of social behavior” (n=2, 1.8%); and “other aims” (n=16, 14.7%)—for example, “information” (n=3, 2.8%) or “education” (n=2, 1.8%; Table 2).

**Table 1:** Frequency of the app store categories of the mobile health apps for gastrointestinal disorders (multiple selection possible).

App store category	App (N=109), n (%)
Parenting	1 (0.9)
Medical	33 (30.3)
Lifestyle	3 (2.8)
Health and fitness	76 (69.7)
Food and drinks	11 (10.1)
Entertainment	3 (2.8)
Education	2 (1.8)
Books and references	2 (1.8)

**Table 2:** Frequency of the aims of the mobile health apps for gastrointestinal disorders (multiple selection possible).

Aim	App (N=109), n (%)
Improvement of general well-being	92 (84.4)
Promotion of physical health	86 (78.9)
Entertainment	3 (2.8)
Support for behavioral changes	33 (30.3)
Support in achieving individual goals	27 (24.8)
Reduction of stress	7 (6.4)
Reduction of fear	4 (3.7)
Improvement of social behavior	2 (1.8)
Other aims	16 (14.7)

### Content and Functions

Of the 109 mHealth apps, almost all (n=91, 83.5%) focused on educational information about gastrointestinal diseases; over half (n=71, 65.1%) offered specific “tips and advice”; and the following methods were also frequent: “monitoring and tracking” (n=22, 20.2%), “alternative medical intervention elements” (n=18, 16.5%), “data collection and measurement” (n=13, 11.9%), feedback (n=13, 11.9%), and “memory, reminder, and amplifier” (n=7, 6.4%). The frequency of the methods used is summarized in Table 3.

Almost all mHealth apps (n=101, 92.7%) had “treatment” as their field of application. Other frequent fields were “prevention of disease” (n=73, 67%), “rehabilitation” (n=51, 46.8%), and “aftercare” (n=45, 41.3%).

**Table 3:** Frequency of methods in the included mobile health apps for gastrointestinal disorders (multiple selection possible).

Method	App (N=109), n (%)
Information and education	91 (83.5)
Tips and advice	71 (65.1)
Monitoring and tracking	22 (20.2)
Alternative intervention elements	18 (16.5)
Data collection and measurement	13 (11.9)
Feedback	13 (11.9)
Memory, reminder, and amplifier	7 (6.4)
Pursuing own goals	5 (4.6)
Traditional medicine	4 (3.7)
Strategies, skills, and training	2 (1.8)
Relaxing exercises	2 (1.8)
Gamification	2 (1.8)
Tailored interventions and real-time feedback	2 (1.8)
Other	1 (0.9)
Physical exercises	1 (0.9)
Mindfulness and gratefulness	1 (0.9)
Acceptance	1 (0.9)

### Privacy and Security Features

Of the 109 mHealth apps, 9 (8.2%) had no privacy and security features; 69 (63.3%) had an imprint, and 54 (49.5%) had a visible privacy policy; 16 (14.7%) required consent to data collection in an active form, and 54 (49.5%) in a passive form; and 11 (10.1%) ensured the security of data transfer, 11 (10.1%) required a log-in, 13 (11.9%) offered a password protection system, 7 (6.4%) informed about the conflicts of interests or financial background, and 1 (0.9%) had an emergency function.

### Quality Rating

The overall quality of mobile health apps was average (mean 2.90, SD 0.52; ranging from 1.84 to 4.47). The top 10 ranked mHealth apps with the highest overall quality are listed in Tables 4 and 5. Concordance between raters was good to excellent (ICC from 0.76, 95%CI 0.70-0.81 to 0.93, 95% CI 0.92-0.94).

The average quality ratings of all included mHealth apps of the MARS subscales were the following: engagement, 2.47 (SD 0.74; range 1.10-5.00); functionality, 4.08 (SD 0.57; range 2.25-5.00); aesthetics, 3.19 (SD 0.76; range 1.17-4.83); and information quality, 1.89 (SD 0.66; range 0.57-3.79). The subjective quality was 2.16 (SD 0.79; range 1.00-4.50) and the perceived impact was 2.33 (SD 0.63; range 1.15-4.08; Table 6).

**Table 4:** Top 10 ranked mobile health apps according to Mobile Application Rating Scale overall quality, target, developer, and category.

App	Rating, mean	Target^a^	Developer	Category^b^
vyoapp - Die CED-App	4.47	Digestive problems	Takeda Pharma Vertriebs GMbH & Co. KG	Medical
My IBD Manager from AGA	4.18	Ulcerative colitis	@Point of care	Health and fitness
MyColitis	4.05	Ulcerative colitis	myColitis	Health and fitness
My IBD Care	3.86	Inflammatory bowel disease	Ampersand health limited	Medicine and health and fitness
Cliexa-IBD	3.85	Inflammatory bowel disease	CN4CE, Inc	Medical
Poop Tracker – Toilet Login	3.82	Digestive problems	Appstronaut Studios	Health and fitness
Doc4Me – IBD Doctor Search	3.71	Inflammatory bowel disease	The North American Society for Pediatric Gastroenterology, Hepatology and Nutrition and Gotomo GmbH	Medical and health and fitness
Food Navi – Coeliac	3.71	Celiac disease	Goe GmbH	Health and fitness and food and drink
Histamin, Fructose & Co.	3.69	Food intolerance	Baliza GmbH	Health and fitness and food and drink
Reflux Tracker	3.68	Digestive problems	Gotomo GmbH	Health and fitness

^a^Target disease or search term.

^b^Category in the Apple App or Google Play store.

**Table 5:** Privacy policy, informed consent, certification, and price of the top 10 ranked mobile health apps.

App	Privacy policy^a^	Informed consent^b^	Certification^c^	Price, € (US $)
vyoapp - Die CED-App	Yes	No	No	0 (0)
My IBD Manager from AGA	Yes	Yes	American Gastroenterological Association	0 (0)
MyColitis	Yes	No	No	0 (0)
My IBD Care	Yes	Yes	No	0 (0)
Cliexa-IBD	Yes	Yes	No	0 (0)
Poop Tracker – Toilet Login	Yes	No	No	0 (0)
Doc4Me – IBD Doctor Search	Yes	Yes	No	0 (0)
Food Navi – Coeliac	No	No	No	3.49 (4.24)
Histamin, Fructose & Co.	Yes	No	No	5.99 (7.27)
Reflux Tracker	No	No	No	0 (0)

^a^Mobile health app had a privacy policy that could be accessed.

^b^Informed consent was actively obtained.

^c^Mobile health app was certified or developed under professional surveillance.

**Table 6:** Subjective quality rating and the rating of perceived impact on user according to the Mobile Application Rating Scale.

Variable	Rating, mean (SD)
Subjective quality rating	2.35 (0.84)
Recommendable	2.17 (0.94)
Probability of using the app in the next 12 months	2.53 (1.06)
Payment	1.31 (0.58)
Star rating	2.63 (0.89)
Perceived impact	2.31 (0.64)
Increased awareness	2.46 (0.90)
Increased knowledge	2.60 (1.00)
Attitudes	2.14 (0.65)
Fosters intention to change	2.10 (0.83)
Empowers help-seeking behavior	2.22 (1.17)
Fosters behavior change	2.49 (0.83)

### Quality Rating on Evidence

Only 2 (1.8%) of the 109 mHealth apps were certified and developed in concordance with guidelines published by the American Gastroenterological Association. None of the mHealth apps had an evidence base.

### Correlation Patterns

The user star rating did not correlate with the MARS overall score or the individual subdimensions (overall: *r*=–0.03; *P*=.86; engagement: *r*=–0.11; *P*=.46; functionality: *r*=–0.17; *P*=.23; aesthetics: *r*=0.15; *P*=.28; information: *r*=0.02; *P*=.87; subjective quality: *r*=0.07; *P*=.61; perceived impact: *r*=–0.12; *P*=.39).

## Discussion

### Principal Findings

This study is the first that comprehensively and systematically reviewed mHealth apps for different gastrointestinal disorders available in the Google Play and Apple App stores [39]. The quality of the mHealth apps was investigated by standardized expert ratings using the MARS-G [48]. In total, 109 mHealth apps with a focus on gastrointestinal disorders were included. Therefore, this analysis offers the first comprehensive systematic expert review of mHealth apps in the field of gastroenterology.

The majority of the mHealth apps were found in the categories “health and fitness” and “medical.” The average quality of the included apps was moderate, according to the applied quality criteria. Only 2 mHealth apps were certified and developed in concordance with approved guidelines such as those from the American Gastroenterological Association. This fact is alarming because the concordance of a mHealth app with approved guidelines is crucial to prevent mistreatment and misinformation. A similar lack of adherence to well-established medical guidelines was found in mHealth app quality reviews for depression and posttraumatic stress disorder [57,62]. Moreover, our data show that user star ratings did not correlate with the experts’ MARS ratings. However, this finding is in accordance with a previous study on mHealth apps for posttraumatic stress disorder and in contradiction to a systematic review of mHealth apps for mindfulness [59,67]. These findings underline the need for systematic reviews to empower patients and health care providers in informed health care decisions. Freely available platforms, which display expert quality ratings of mHealth apps such as the MHAD [61], Psyberguide [68], or KVAppradar [69], have been installed as a possible solution to empower patients and health care providers. In addition to these platforms that offer an evaluation of available mHealth apps based on the general criteria of scientific evidence, professional gastroenterological societies should participate in the development and assessment of mHealth apps in consideration of established guidelines. Regarding the rapid progress in the methods of disease monitoring and therapy of gastrointestinal disorders, suitable apps should be constantly updated for adequate support. In particular, for long-term gastrointestinal disorders, such as IBD, which are characterized by an unstable disease course with recurrent remission and exacerbation, mHealth apps could be a promising approach for symptom monitoring with an early detection of disease relapse. As previous studies have shown that self-reporting symptom diaries correlate with disease activity index for Crohn disease [70,71], validated symptom assessment questionnaires could be implemented in future mHealth apps.

From the patients and health care providers’ perspectives, mHealth interventions could demonstrate a great potential to facilitate the monitoring of symptoms, improve self-management–related physical or psychosocial consequences, and maintain compliance [72-77]. Rapid advancement in mobile technology may enable real-time data capture and exchange between patient self-monitoring devices and a remote monitoring system, which creates promising opportunities to provide prompt feedback to patient-generated alerts and specific needs [38].

Besides the lack of mHealth apps for adequate symptom monitoring, our results showed that none of the evaluated apps were designed to evaluate adverse drug reactions that occur during disease therapy. Giraud et al [78] have demonstrated that 40.9% (N=1179) of patients with IBD that participated in the IBDREAM registry had at least 1 adverse drug reaction, and 24 new adverse drug reactions were found based on their analysis. These findings suggest that the evaluation of adverse events during maintenance therapy in IBD and possibly other gastrointestinal diseases should be monitored closely to timely change or adapt drug dose or substance choice for individual-tailored therapy. The use of mHealth apps for the monitoring of adverse drug reactions, especially during the start of a new therapeutical agent, could be a new field for the implementation of mHealth apps in clinical practice. The clinical monitoring of disease activity and drug compatibility could be further enhanced by wearable devices that track physical parameters and by noninvasive biomarker monitoring (eg, c-reactive protein or interleukin-1 for IBD from sweat [79]). In their comprehensive review, Chong and Woo [80] have demonstrated that approaches for the implementation of wearable sensor systems for gastrointestinal disease already exist and could change clinical practice in the near future [80].

Furthermore, the results highlight the need for a comprehensive evaluation of clinical effectiveness and economic effects. In particular, the long-term effects and cost-effectiveness of mHealth apps to manage gastrointestinal diseases should be elaborated in future studies [38]. Currently, studies that have evaluated the cost-effectiveness of telemedicine-directed treatment and monitoring of IBD show a reduction of hospitalization and therapy costs [81] but remain controversial regarding the total cost-effectiveness of telemedical interventions [82]. Since the use of biologicals has been identified as the major cost driver for IBD [83], mHealth apps could help to early de-escalate and optimize biological treatment after constant disease remission and enhance conventional therapy admission to prevent unnecessary therapy escalation to expensive biologicals. To date (2022), in Germany, a central register for Conformité Européenne–certified eHealth apps with scientifically proven benefit for patients has been established. Apps that are listed in the national digitale Gesundheitsanwendungen (digital health care app in German) register are prescriptible by health care professionals. The costs could be reimbursed by the patient’s health insurance companies, which might be a step toward the implementation of trustworthy, certified apps into daily health care.

Additionally, the review revealed that data security is not always guaranteed when using mHealth apps. As health data are highly sensitive, this lack of guarantee is one reason why the use of mHealth apps in the management of gastrointestinal diseases cannot be clearly recommended currently. We found that the security of data transfer was only ensured in 14% of the mHealth apps. As patient safety is paramount, data security is a keystone for adopting mobile technologies into health care. In this field, respect for privacy, security, the disclosure of data sharing, traceability, and the guarantee of transparency are essential factors. These factors are in line with other reviews of the data security and privacy of mHealth apps for smoking cessation, depression, and older adults [40,41,46].

When using and implementing mHealth technologies into health care systems, it will be important to know how these technologies will fit within the existing organizational framework, which may involve changes in business structure and culture, workflow, and staff. In this context, the primarily legal aspects of mHealth app use play a substantial role. National regulations for mHealth approaches such as the act on medical devices—the Medical Devices Directive—for the European Union or the Food and Drug Administration regulation body for the United States exist. The harmonization of the regulation instruments is crucial for the sufficient uptake of mHealth solutions worldwide. Such worldwide standards for the safe use of mHealth apps in gastroenterology should include (1) being based on current standards and medical guidelines, (2) randomized controlled trial testing for effectiveness, (3) high standards for data security, and (4) minimal and economic data recording.

We acknowledge several limitations regarding this review. First, due to the rapid growth and dynamic changes in mHealth apps available on the global market, this study can only represent a snapshot view of the available mHealth apps as of July 2021 for the management of gastrointestinal disorders. The continuous monitoring of the market is mandatory to reliably inform users and health care providers. Second, the main focus was on English- and German-language medical mHealth apps, which might have impaired the generalizability of the results, as the quality of mHealth apps may vary between countries and continents. Third, the review included all types of gastrointestinal disorders with a focus on inflammatory and nutritive bowel diseases. An even more precise analysis of mHealth apps addressing the multiple subspecialties of gastrointestinal disorders could be promising. Furthermore, the analysis of mHealth apps for hepatobiliary disease and gastrointestinal cancer (eg, mHealth apps for the patient-related surveillance of adverse events due to chemotherapy) should be evaluated specifically in further studies. Fourth, the user star ratings in the app stores may refer to various versions of an mHealth app and are aggregated across the different versions. Therefore, the MARS rating and the user star rating could refer to different versions.

### Conclusion

This systematic review of mHealth apps that manage gastrointestinal diseases found a moderate overall quality of mHealth apps available in app stores. The quality of user engagement and information quality was rated as poor, thus limiting the possible positive effects of mHealth app use to manage gastrointestinal diseases. Furthermore, data safety and privacy were mostly not given. Moreover, there were no efficacy studies on the included mHealth apps, and only 2 mHealth apps were following well-established guidelines for the treatment of gastrointestinal diseases. Taken together, these findings implicate a red flag of the use of currently available mHealth apps for the management of gastrointestinal diseases. Nevertheless, given the possible positive impact of mHealth apps in the routine care of individuals with gastrointestinal diseases, an improvement in the quality of medical content for mHealth apps and data safety is mandatory.

## Abbreviations

IBD: inflammatory bowel disease
ICC: intraclass correlation
IRR: interrater reliability
MARS: Mobile Application Rating Scale
MARS-G: German version of the Mobile Application Rating Scale
MHAD: Mobile Health App Database
mHealth: mobile health
PRISMA: Preferred Reporting Items for Systematic Reviews and Meta-Analyses

## Data Availability

The primary data of the systematic review can be provided by the corresponding author on a reasonable request. Data will only be shared for scientific purposes. Data sharing agreements may have to be signed depending on the request.
